# Supplementary material for: The Endothelial Landscape and Its Role in Von Hippel–Lindau Disease
Source: Cells. 2021 Sep 4;10(9):2313. doi: 10.3390/cells10092313 (PMC8465092; doi:10.3390/cells10092313)
Supplement: Supplementary file 1 [file cells-10-02313-s001.zip › cells-1338457-supplementary.pdf]

Supplementary Table S1: Gene expression pattern of selected genes of interest (all $p < 0.05$) in logCPM units, as seen by differential expression analysis from RNA sequencing.

					LogCPM								
					Control		VHL						
	**Protein**	**Gene**	**p value**	**adjusted p value**	**C2**	**C3**	**V1**	**V2**	**V3**	**V4**	**V5**	**logFC**	**Up/Down**
Cell adhesion	CD44	CD44	0.0065	0.2795	8.74	8.74	5.19	7.23	7.74	7.07	5.39	-2.20	Down
	Intercellular Adhesion Molecule 1 (ICAM-1)/CD54	ICAM1	0.0001	0.0448	6.75	7.13	3.66	3.52	4.79	4.49	5.31	-2.68	Down
	Vascular cell adhesion protein 1 (VCAM-1)/CD106	VCAM1	0.0023	0.1855	6.03	4.56	0.61	0.56	2.06	2.44	4.87	-3.67	Down
	Intercellular Adhesion Molecule 1 (ICAM-1)	ICAM1	0.0001	0.0448	6.75	7.13	3.66	3.52	4.79	4.49	5.31	-2.68	Down
	Fibronectin	FN1	0.0153	0.3740	11.90	12.44	10.88	10.17	10.14	12.31	10.79	-1.48	Down
	Endosialin/CD248	CD248	0.0358	0.4720	1.60	1.39	0.02	0.02	0.02	1.08	1.12	-2.05	Down
	Disintegrin and metalloproteinase domain-containing protein 15 (ADAM15)	ADAM15	0.0183	0.3935	8.48	8.63	9.90	9.41	9.47	8.98	8.93	0.75	Up
	Endomucin	EMCN	0.0099	0.3309	6.87	6.18	8.36	7.13	7.67	8.95	9.26	1.74	Up
Signal transduction	Vascular endothelial growth factor receptor 2 (VEGFR-2)/KDR/Flk-1/CD309	KDR	0.5164	0.9077	7.79	7.62	8.19	7.84	7.96	8.26	7.17	0.20	Up
	Interleukin 13 receptor, alpha 1 (IL13RA1)/CD213A1	IL13RA1	0.0297	0.4471	7.47	7.79	6.38	6.09	6.08	7.64	6.57	-1.09	Down
	Endothelial protein C receptor (EPCR)/CD201	PROCR	0.0060	0.2747	7.25	6.12	7.61	8.43	8.25	7.46	7.84	1.24	Up
	RalA-binding protein 1 (RALBP1)	RALBP1	0.0436	0.5077	7.46	7.48	7.84	7.76	8.04	7.94	8.47	0.53	Up
	Anthrax toxin receptor 1 (ANTXR1)/TEM8	ANTXR1	0.0000	0.0286	8.01	7.88	4.83	2.79	3.04	5.42	3.65	-4.13	Down
	Serum deprivation-response protein (SDPR)/Cavin2	CAVIN2	0.0001	0.0324	6.41	5.70	9.18	8.33	7.89	8.19	9.01	2.47	Up
	Endoglin (ENG)	ENG	0.0495	0.5221	11.21	11.38	10.68	9.61	9.57	10.86	10.78	-0.93	Down

	Fibroblast growth factor receptor 4/CD334	FGFR4	0.0632	0.5632	2.71	2.41	0.61	2.35	1.50	0.02	1.73	-1.69	Down
	Platelet-derived growth factor subunit A	PDGFA	0.0236	0.4261	6.97	6.96	4.75	6.55	6.11	6.38	5.80	-1.05	Down
Immune response	Tumor necrosis factor, alpha-induced protein 3/A20	TNFAIP3	0.0005	0.0872	4.86	5.37	1.83	2.89	0.94	3.77	2.76	-2.96	Down
	Interleukin 1 beta (IL-1β)	IL1B	0.0000	0.0286	3.65	4.31	0.61	0.02	0.02	0.02	1.12	-4.97	Down
	Interleukin 6 (IL-6)	IL6	0.0106	0.3357	5.00	3.68	2.48	2.07	3.04	2.28	3.65	-1.74	Down
	Chemokine (C-C motif) ligand 20 (CCL20)	CCL20	0.0001	0.0448	4.01	3.80	0.02	0.95	0.94	1.08	1.73	-3.76	Down
	Tumor necrosis factor (TNF)	TNF	0.0016	0.1583	1.60	3.18	0.02	0.02	0.02	1.08	0.02	-3.54	Down
	Prion protein (PrP)/CD230	PRNP	0.0033	0.2113	7.84	7.12	6.07	5.75	6.32	6.83	6.32	-1.26	Down
Metabolism	Fatty-acid-binding proteins (FABP)	FABP5	0.0013	0.1447	5.33	3.85	7.38	8.69	6.64	6.98	6.95	2.83	Up
		FABP4	0.0157	0.3740	4.05	3.49	7.93	10.87	5.37	7.96	9.02	4.63	Up
	Hexokinase 2	HK2	0.0861	0.6182	1.82	2.26	3.77	3.73	4.22	2.44	2.34	1.57	Up
	Pyruvate kinase isozymes M1/M2	PKM	0.0472	0.5209	9.76	8.71	10.52	10.45	10.68	9.79	9.23	0.95	Up
ROS	Endothelial NOS (eNOS)/Nitric oxide synthase 3 (NOS3)	NOS3	0.0329	0.4576	2.18	3.00	6.23	3.58	6.69	6.21	5.88	3.39	Up
	Superoxide dismutase 2	SOD2	0.0000	0.0290	8.35	7.94	5.68	5.28	5.85	3.99	5.16	-2.97	Down
	Thioredoxin reductase 1	TXNRD1	0.0023	0.1855	9.50	10.28	8.34	8.00	9.21	8.23	8.28	-1.46	Down
	thioredoxin interacting protein	TXNIP	0.0255	0.4319	6.60	6.65	4.95	6.31	2.35	4.72	5.11	-2.01	Down
	Glutathione reductase (GR)	GSR	0.0240	0.4261	6.48	6.61	5.72	6.34	6.21	5.76	5.80	-0.59	Down
	Glutathione peroxidase (GPx)	GPX1	0.0547	0.5379	9.92	8.96	9.93	9.99	10.14	9.76	10.59	0.63	Up
	Cathepsin B	CTSB	0.0646	0.5637	11.50	11.39	10.47	10.81	10.42	11.30	10.94	-0.62	Down
	Prostaglandin-endoperoxide synthase 2/COX-2	PTGS2	0.0324	0.4551	4.81	5.29	2.72	0.02	3.51	4.08	4.33	-2.69	Down
	Cytochrome b-245, alpha polypeptide	CYBA	0.0253	0.4300	8.51	7.62	6.27	7.06	6.31	7.20	7.83	-1.15	Down

	NAD(P)H dehydrogenase, quinone 1	NQO1	0.0150	0.3719	10.85	9.96	8.88	7.45	9.19	6.48	9.16	-2.14	Down
	Sulfiredoxin 1 homolog	SRXN1	0.0133	0.3572	1.82	2.79	1.02	0.56	1.25	0.65	0.67	-1.91	Down
Transcription regulation	Polymerase I and transcript release factor/Cavin1/PTRF	CAVIN1	0.0152	0.3733	10.29	9.72	11.24	10.78	10.93	10.26	10.61	0.80	Up
	B-cell lymphoma 6 protein	BCL6	0.0297	0.4472	5.00	4.75	4.19	3.78	4.45	2.82	3.95	-1.05	Down
	B-cell CLL/lymphoma 6 member B protein	BCL6B	0.0033	0.2100	6.30	5.37	7.50	7.62	7.77	6.45	7.13	1.48	Up
	B-cell CLL/lymphoma 9 like	BCL9L	0.0510	0.5261	4.05	4.46	3.88	3.96	3.19	3.03	2.50	-0.92	Down
	B-cell lymphoma/leukemia 10	BCL10	0.0079	0.3031	5.86	5.33	6.13	6.91	6.44	6.46	7.07	1.04	Up
	Hypoxia-inducible factor 3 alpha	HIF3A	0.0362	0.4729	5.40	5.92	3.83	5.53	5.23	2.82	2.50	-1.64	Down
	Forkhead box proteins	FOXO4	0.1236	0.6604	2.59	2.68	3.72	3.20	3.68	2.70	3.09	0.84	Up
		FOXF1	0.0011	0.1432	5.68	5.44	1.02	3.34	3.77	0.02	2.50	-3.82	Down
		FOXL1	0.0413	0.4987	2.47	4.69	2.60	1.72	2.21	2.11	1.73	-1.57	Down
		FOXD2-AS1	0.0617	0.5610	1.82	2.68	1.34	0.95	1.25	1.68	1.12	-1.16	Down
		FOXJ2	0.0738	0.5825	4.62	4.77	3.72	4.57	4.26	4.30	3.90	-0.51	Down
	RNA binding motif protein 9 (RBM9)	RBFOX2	0.0168	0.3822	6.57	6.01	7.14	7.04	7.00	6.77	6.74	0.64	Up
	SOX transcription factors	SOX18	0.0047	0.2478	6.43	6.62	10.02	9.04	8.42	7.03	9.01	2.16	Up
		SOX17	0.0119	0.3482	6.41	6.60	6.14	5.36	5.57	5.40	5.92	-0.86	Down
		SOX7	0.0793	0.6002	5.86	5.48	6.42	5.81	7.21	5.98	6.96	0.81	Up
	Excision repair cross-complementation group 2 (ERCC2)	ERCC2	0.0072	0.2937	7.40	7.58	6.32	6.10	6.82	6.68	6.86	-0.95	Down
	Fanconi anemia, complementation group C	FANCC	0.0342	0.4630	2.91	3.18	4.06	4.16	3.62	3.59	3.65	0.94	Up
	Caveolae-associated protein 3	CAVIN3	0.0093	0.3284	5.41	4.05	7.24	6.16	5.92	5.84	6.23	1.60	Up
Cell homeo	FAD-dependent oxidoreductase domain-containing protein 2	FOXRED2	0.0974	0.6339	4.53	3.96	3.41	3.96	3.87	3.52	2.76	-0.68	Down

	Matrix metalloproteinases	MMP16	0.0144	0.3662	0.02	0.02	3.41	4.63	3.39	1.68	2.88	5.08	Up
		MMP1	0.0990	0.6339	6.80	3.74	10.03	9.02	12.20	10.07	3.28	3.77	Up
		MMP19	0.0069	0.2878	2.59	3.10	0.02	1.72	0.94	1.91	0.02	-2.70	Down
	Kinesin family member 9	KIF9	0.0820	0.6059	3.59	3.34	4.06	4.04	3.77	4.08	4.66	0.76	Up
	Tropomodulin 1	TMOD1	0.0374	0.4791	1.60	2.55	0.61	1.50	0.55	0.65	1.12	-1.55	Down
	Nucleoredoxin	NXN	0.0014	0.1502	2.71	2.79	6.92	7.24	5.13	6.12	6.42	3.84	Up

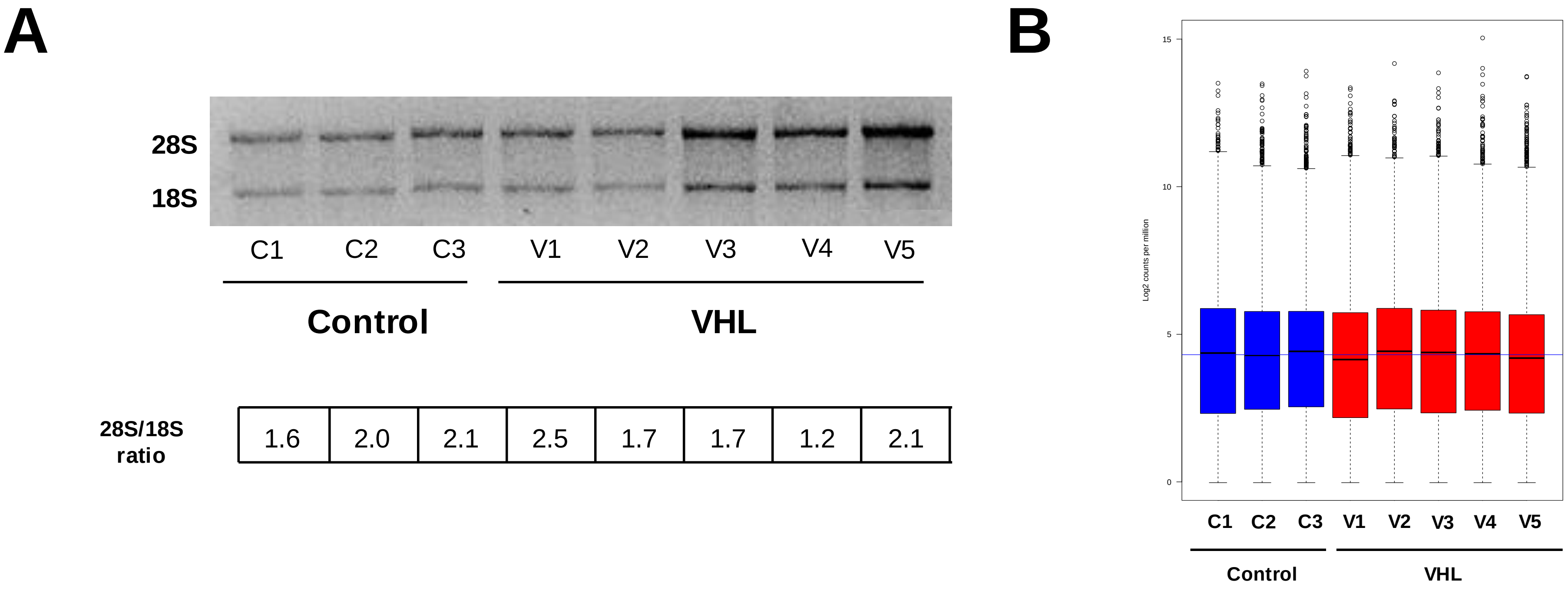

Supplementary Figure S1. A) RNA gel electrophoresis to check RNA quality and integriy. First band corresponds to 28S ribosomal RNA, while the second corresponds to 18S ribosomal RNA. 28S/18S ribosomal RNA ratio is displayed underneath. **B)** Boxplot representation of RNAseq's logCPM (log Count per Million).
